# Genomic epidemiology reveals geographically structured co-circulation of AFR10 and AFR13 Vibrio cholerae lineages driving cholera outbreaks in Tanzania (2022–2024)

**DOI:** 10.1099/mgen.0.001813

**Published:** 2026-08-18

**Keywords:** cholera, genomic epidemiology, T10/AFR10, T13/AFR13, Tanzania, whole-genome sequencing

## Abstract

Cholera remains a persistent public health challenge in Tanzania, with recurrent outbreaks across multiple regions overwhelming the health systems. Despite the ongoing surveillance to control the transmission, limited genomic characterization of circulating *Vibrio cholerae* strains has constrained traceability of outbreak sources and transmission dynamics including virulence determinants and antimicrobial resistance profiles to guide clinical and public health interventions. We conducted a genome analysis of 144 archived isolates from 2022 to 2024 cholera outbreaks from 16 regions across Tanzania. Sequencing was performed using Oxford Nanopore platforms. Comprehensive bioinformatics analysis was applied including genome assembly, multi-locus sequence typing, resistance gene detection, virulence profiling and phylogenetic reconstruction. Of 144 isolates, sequencing confirmed 119 to be *V. cholerae*, of which 109 (91.6%) belonged to sequence type ST69. Phylogenetic analysis identified two distinct transmission lineages: AFR10 (formerly T10) and AFR13 (formerly T13), demonstrating marked geographic stratification. AFR10 isolates clustered predominantly around Lake Tanganyika basin, bordering the Democratic Republic of the Congo and Zambia, whereas AFR13 isolates were widely distributed across different zones of Tanzania. All isolates harboured key virulence genes (*ctxA, ctxB, tcpA*) and multiple resistance genes conferring resistance to co-trimoxazole and macrolides. The *catB gene* was found to be silenced in these isolates. Notably, tetracycline resistance genes (*tetA/B/C*) were absent. The *qacEdelta* gene was only detected in AFR13 isolates and has been linked to resistance to quaternary ammonium compounds (antiseptics and disinfectants) in Gram-negative bacteria from other studies. These findings demonstrate the co-circulation of two sublineages (AFR10 and AFR13) of the seventh pandemic El Tor lineage with distinct geographic distribution, suggesting both localized persistence and regional circulation. The observed genotypic and phenotypic susceptibility to tetracycline supports the continued use of doxycycline as a first-line therapy in adults. However, the detected macrolide resistance (an alternative treatment for children) and resistance genes associated with reduced susceptibility to antiseptics underscore the need for extensive monitoring. Strengthened national and cross-border genomic surveillance is essential for effective cholera control.

**Impact Statement**

This study is among the first to provide nationwide genomic insights into *Vibrio cholerae* driving recent cholera outbreaks in Tanzania, addressing a surveillance gap since 2017. Analysis of isolates from 16 regions representative of all Tanzania zones has demonstrated how two co-circulating seventh-pandemic lineages (AFR10 and AFR13) shape both localized persistence and widespread transmission. The work accentuates the importance of genomic and bioinformatics tools in better understanding of transmission patterns beyond conventional surveillance. In this study, the tools have enabled identification of geographic clustering and regional relatedness across shared ecological corridors, including the Lake Tanganyika basin.

The findings have broadened utility for public health, clinical management and regional disease control programmes. Evidence of widespread multidrug resistance, alongside preserved susceptibility to tetracyclines, directly informs treatment guidelines, while the detection of disinfectant resistance raises important considerations for infection prevention control strategies.

Overall, this study represents a significant step in advancing integration of genomic, geographic and antimicrobial resistance data to generate actionable insights for timely outbreak response. It also underscores the importance of sustained genomic surveillance in cholera-endemic settings. These findings are highly relevant to other countries with similar transmission dynamics across sub-Saharan Africa and beyond.

## Data Summary

 All genomes generated have been deposited in the European Nucleotide Archive (ENA; BioProject PRJEB110494), and individual accession numbers are listed in Table S1, available in the online Supplementary Material. All supporting data have also been provided within the article or in the supplementary data files.

## Introduction

Cholera, caused by toxigenic *Vibrio cholerae* serogroup O1, remains a major global health threat. It is estimated to cause 2.86 million cases and 95,000 deaths annually in endemic countries [1]. Sub-Saharan Africa bears a disproportionate burden, accounting for ~60% of global cases [1]. Between 2000 and 2023, the WHO African Region reported 2,727,172 cholera cases and 63,182 deaths from 44 of the 47 countries in the region [2]. Persistent hotspot regions have been documented in Central, Eastern and Western Africa [2]. The continued high incidence is driven by inadequate water and sanitation infrastructure, limited access to healthcare, socioeconomic challenges and weak surveillance systems in many affected countries [3].

Cholera was first reported in Tanzania in 1974 along the shores of Lake Nyasa (also called Lake Malawi in Malawi or Lake Niassa in Mozambique) [4, 5]. Since then, the country has experienced recurrent outbreaks and has recorded more than 250,000 cases and 13,000 deaths over the past five decades [6]. The most recent outbreak began on 5 September 2023, affecting 13 regions, resulting in 1,592 cases and 34 deaths (case fatality rate [CFR] 2.2%) [7]. Since January 2024, outbreaks have been reported in 24 of the 26 Tanzania mainland regions, excluding Njombe and Kilimanjaro. According to the Ministry of Health situation report of 8 December 2025, a cumulative total of 16,536 cases and 205 deaths (CFR 1.2%) had been recorded.

Across Tanzania and the wider East African region, cholera disproportionately affects fishing communities and agricultural workers. A retrospective analysis of isolates from the 2015–2016 cholera outbreak in Mwanza identified peasants (33%) and fishermen (22.6%) as the most affected groups among 352 laboratory-confirmed cases [3]. Reliance on untreated water sources, poor sanitation, overcrowding and other behavioural and environmental risk factors continue to drive transmission in many low- and middle-income countries [8]. Similar factors have also been identified in Tanzania as contributors to cholera persistence. For example, outbreaks have been linked to environmental reservoirs associated with large lakes, inadequate water, sanitation and hygiene services, urban crowding, recurrent transmission in lakeshore communities and gaps in surveillance and health system capacity [8–10].

Several studies have investigated the epidemiology of cholera in Tanzania using routinely available laboratory methods, including serological characterization. One study that analysed the 2016–2017 cholera outbreak isolates across 11 of the 26 regions of Tanzania mainland identified *V. cholerae* serogroup O1 serotype Ogawa as the predominant serotype accounting for 98% of isolates [11]. However, serological data alone provide limited insight into transmission pathways and do not capture pathogen evolution or antimicrobial resistance (AMR) dynamics.

Molecular epidemiology has substantially advanced understanding of cholera transmission and evolution. The seventh pandemic El Tor (7PET) lineage, which emerged in 1961, has diversified into multiple transmission sublineages, with at least 17 African regional sublineages (AFR1–AFR17) identified to date [12, 13]. These sublineages correspond to distinct epidemic waves, geographic distributions and AMR profiles. In Africa, the 7PET lineage has been introduced multiple times from Asia since 1970, with multidrug-resistant sublineages becoming dominant after 2000 [12]. Recent genomic studies across East and Central Africa have documented concurrent circulation of sublineages AFR10 and AFR13 lineages, often linked to cross-border transmission and environmental reservoirs such as the African Great Lakes [13–15].

Previous genomic studies in Tanzania identified temporal shifts in circulating lineages. Early outbreaks between 1993 and 1997 were attributed to 7PET sublineage T5 (now AFR5) strains carrying the *ctxB3* allele, while T10 (now AFR10) strains carrying *ctxB1* predominated from 1998 to 2015 [6]. Following 2014, T13 (now AFR13) strains carrying the *ctxB7* gene emerged and became increasingly prevalent [6]. The study also documented deletions in the SXT integrative conjugative element and variations in cholera toxin genes, suggesting ongoing evolution under selective pressures. However, despite the recurrent cholera outbreak waves, no comprehensive genomic surveillance has been undertaken since 2017. Consequently, significant gaps remain in our understanding of the current evolution and diversity of the circulating strains, AMR profiles and transmission dynamics.

Given Tanzania’s geographic position linking East and Central Africa and evidence of trans-national cholera transmission in the region [1, 13, 14, 16], renewed genomic surveillance is a requisite to guide outbreak interventions, patient management and policy decisions.

This study characterizes the *V. cholerae* strains responsible for the cholera outbreaks in Tanzania between 2022 and 2024 to establish genomic diversity, transmission dynamics and AMR determinants. The findings will inform national cholera control strategies and contribute to regional elimination efforts under the Global Task Force on Cholera Control towards their goal to reduce cholera deaths by 90% by 2030 [17].

## Methods

### Study design and setting

This retrospective observational genomic surveillance study analysed *V. cholerae* isolates collected through Tanzania’s national cholera surveillance programme between January 2022 and December 2024. Isolates were obtained from 16 regions of Tanzania Mainland: Arusha, Dar es Salaam, Dodoma, Geita, Kagera, Katavi, Kigoma, Kilimanjaro, Morogoro, Mwanza, Pwani, Rukwa, Ruvuma, Shinyanga, Simiyu and Singida. These regions encompass diverse ecological and epidemiological contexts, including coastal settings, the lake zone and highland areas, with cross-border connectivity to neighbouring countries. The sequenced isolates provided broad geographic coverage across major epidemiological zones of Tanzania, but they should not be interpreted as a population-representative sample. Inclusion depended on outbreak detection, referral of isolates to NPHL, isolate viability after storage and successful sequencing.

### Sample collection, transportation and bacterial culture

Samples were obtained from patients presenting with acute watery diarrhoea at cholera treatment centres during outbreak periods and from endemic surveillance sites. All cases met the national cholera suspect standard case definition as highlighted by the WHO integrated disease surveillance and response guideline and were laboratory confirmed in the respective facilities. Clinical specimens, either rectal swabs or stool, were then prepared in accordance with the procedure for sample collection, storage and transportation. In the preparation step, stool samples were placed in sterile, leak-proof containers and rectal swabs were immediately transferred into Cary–Blair transport medium to enhance better retrieval of enteric pathogens [18]. Proper labelling and documentation were completed at the point of sample collection, packed in a triple package system and then transported to the testing laboratory at room temperature and finally tested at sentinel laboratories for culture in the respective regions. Culture confirmed *V. cholerae* isolates were then transported to the Tanzania National Public Health Laboratory (NPHL) in Dar es Salaam for retesting and verification. At the NPHL, laboratory processing was conducted in accordance with the procedure for culture of enteric specimens. Samples were cultured on thiosulfate-citrate-bile salts-sucrose (TCBS) agar [19] and incubated at 37 °C for 18–24 h. Presumptive *V. cholerae* colonies (yellow colonies on TCBS) were confirmed using MALDI-TOF MS [20, 21] and serological agglutination with O1 and O139 antisera. Confirmed isolates were stored at −80 °C in broth for subsequent analysis.

### Isolate selection, genomic DNA extraction and sequencing

All available archived isolates labelled as *V. cholerae* and referred to the NPHL biorepository from sentinel and outbreak-response laboratories between 2022 and 2024 were considered for sequencing. No additional case-level sampling was undertaken for this study. Inclusion for sequencing depended on availability in the biorepository, successful recovery after sub-culture, colony morphology and sufficient DNA quantity and quality for library preparation. The archived isolates were sub-cultured on TCBS agar overnight at 37 °C, followed by sub-culture on blood agar (BA) for only isolates that had *V. cholerae* colony morphology (i.e. yellowish colonies) in TCBS agar. Genomic DNA was then extracted from the BA colonies using the QIAamp DNA Mini Kit (Qiagen, Germany) following the manufacturer’s protocol, with a final elution volume of 200 µl. DNA concentration and purity were quantified using a Qubit 4.0 Fluorometer (Thermo Fisher Scientific, USA) according to the dsDNA HS assay protocol and stored at 4 °C prior to library preparation. Sequencing libraries were prepared using Oxford Nanopore Rapid Barcoding Kit (SQK-RBK004) and sequenced on MinION platform using R10.4.1 flow cells at 10× sequencing depth. Basecalling was performed using Dorado v.0.3.4 with super-accurate model.

### Bioinformatics analysis

Quality-filtered reads were processed using the Bactopia v.3.0.1 pipeline implemented in Nextflow, which performs quality control, genome assembly and other downstream analyses [22]. Genome assembly quality was assessed within the Bactopia framework using CheckM to verify expected genome characteristics, including appropriate genome size for *V. cholerae* (~4.0 Mb), G+C content (~47%) and low contamination (<5%). The assembly statistics have been provided in the supplementary materials (SupData05_assembly_report). Multi-locus sequence typing (MLST) was performed with MLST tool (v.2.32.2) [23, 24] to identify sequence type responsible for the outbreaks. AMR genes and virulence factors were assessed using the abricate tool (v.1.2.0) searching through the Comprehensive Antibiotic Resistance Database (CARD) and Virulence Factor Database (VFDB), respectively [25–27], and further validated using assembled genomes uploaded to VibrioWatch which is a curated global platform for *V. cholerae* genomic surveillance in PathogenWatch (https://pathogen.watch/genomes/all?genusId=662&speciesId=666). Transmission lineages and genetic relatedness were determined by phylogenetic comparison with reference genomes representing established 7PET transmission lineages (AFR1–AFR15) [12, 13]. Fast identification and verification of the isolates’ sublineages was done on FASTA files using a customized tool Patho2Clade (https://github.com/IbrahimMauki/Patho2Clade) that employed snippy (https://github.com/tseemann/snippy) for alignment and usher (https://github.com/yatisht/usher) for tree placement with the global reference tree similar to the one implemented by the Vibecheck tool (https://github.com/CholGen/Vibecheck). Plasmid reconstruction and typing were performed using the Mobisuite tool [28], and the plasmid ring image was generated using Brig [29].

### Phylogenetic analysis

For phylogenetic reconstruction, publicly available genomes were obtained from published cholera genomic studies from countries bordering Tanzania (Supplementary data files SupData02–04). To provide contemporary regional context, only sequence data generated within the last 10 years were considered. For each identified BioProject, up to five genomes were selected to minimize overrepresentation of individual studies while maintaining a computationally manageable dataset for the combined phylogenetic analysis. Genome selection was performed by prioritizing recent isolates and limiting inclusion to a maximum of one isolate per region within a country whenever metadata were available. Thereafter, genomes were randomly selected when multiple candidates met these criteria. For sublineage-specific analyses, the initial AFR10 and AFR13 comparison datasets were further supplemented with all publicly available genomes belonging to the respective sublineages from relevant BioProjects.

A reference-independent core-genome approach was selected to avoid potential biases associated with mapping diverse isolates to a single reference genome [30–32]. This approach infers phylogenetic relationships from the conserved core genome rather than a predefined reference strain. Briefly, assembled genomes were annotated using Prokka v.1.15.6 [22] followed by pangenome analysis and core genome alignment with Panaroo v.1.6.0 using default parameters [33]. To minimize the impact of horizontal gene transfer on phylogenetic inference, putative recombinant regions were masked using Gubbins v.3.3.5 [34]. Variable positions were extracted from the core genome alignment using SNP-sites v.2.5.1 [35]. Maximum-likelihood phylogenetic trees were constructed using IQ-TREE 2 v.2.2.6 with a GTR+F+ ASC substitution model selected using the Model Finder Program (-m MFP) according to Bayesian Information Criteria and 1,000 bootstrap replications [36]. All genomes generated in this study have been deposited in the European Nucleotide Archive (ENA) under BioProject accession PRJEB110494. Detailed software parameters and command-line settings used in the analyses are provided in Supplementary File S01 (SupFile_S01_Analysis_Parameters).

## Results

### Geographic and demographic distribution of sequenced samples

**Fig. 1.**
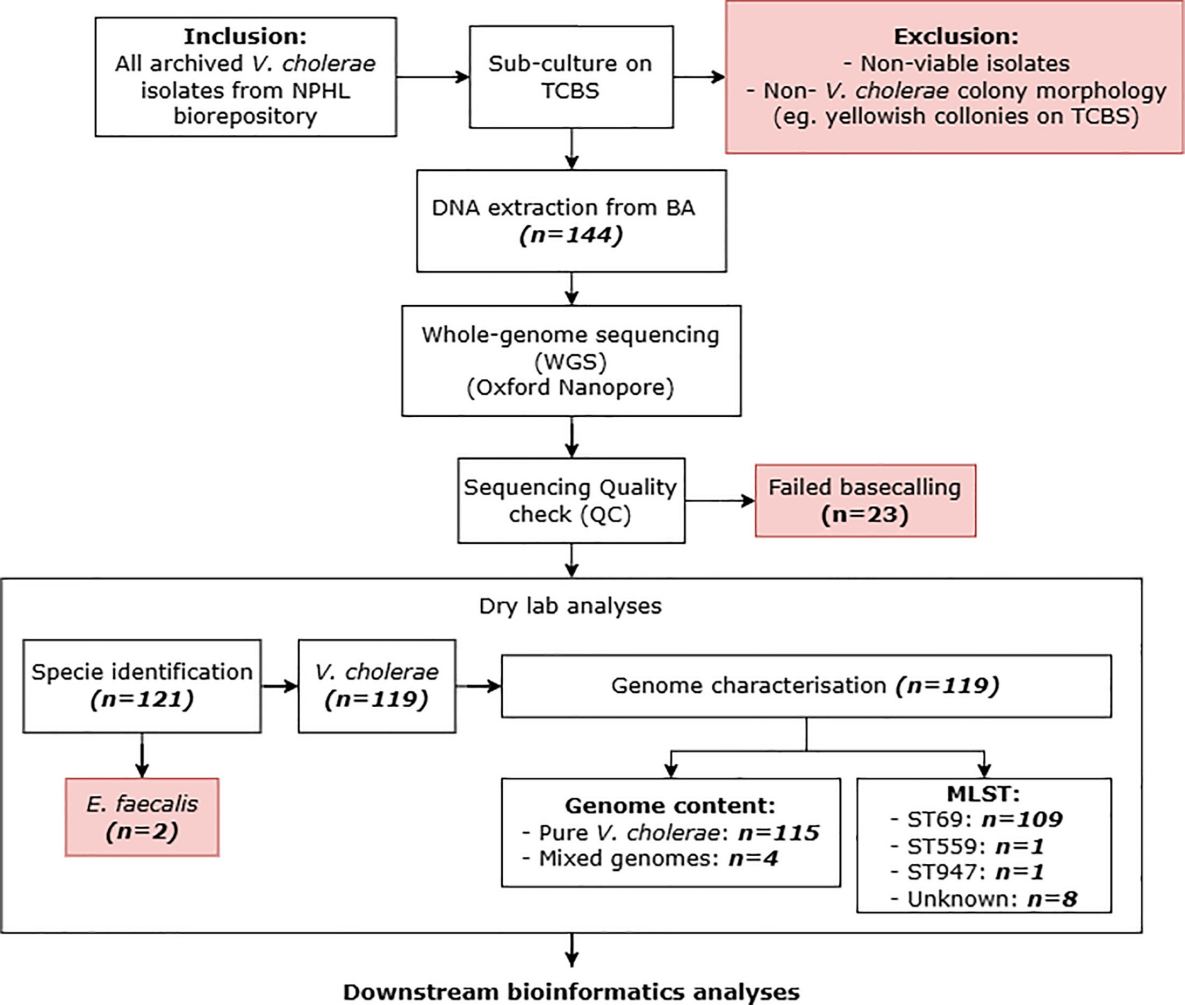
Flowchart describing sequencing QC and exclusion criteria.

A total of 144 viable isolates from the 16 regions were retrieved after sub-culturing of the archived *V. cholerae* isolates, which were then subjected to whole-genome sequencing. Out of these, 121 yielded high-quality sequencing data with 119 samples confirmed to have *V. cholerae* genome, while two were identified as *Enterococcus faecalis*. MLST of the 119 samples identified that 115 had pure *V. cholerae* genomes and 4 had mixed bacterial genomes (see supplementary Data SupData01). The remaining 23 of 144 samples failed during base calling and were excluded from further analysis. Fig. 1 is a flowchart that summarizes the sequencing quality check (QC) steps and exclusion criteria, whereas Table 1 summarizes the geographic distribution of the 119 isolates with *V. cholerae*.

**Table 1.** Geographic distribution of *V. cholerae* isolates (total isolate count=119)

Region	Isolate count	Region	Isolate count
**Arusha**	29	**Singida**	5
**Shinyanga**	16	**Kagera**	4
**Simiyu**	15	**Kilimanjaro**	3
**Dar-es-salaam**	10	**Morogoro**	2
**Katavi**	10	**Mwanza**	2
**Rukwa**	7	**Ruvuma**	2
**Not indicated**	6	**Dodoma**	1
**Pwani**	6	**Kigoma**	1

**Fig. 2.**
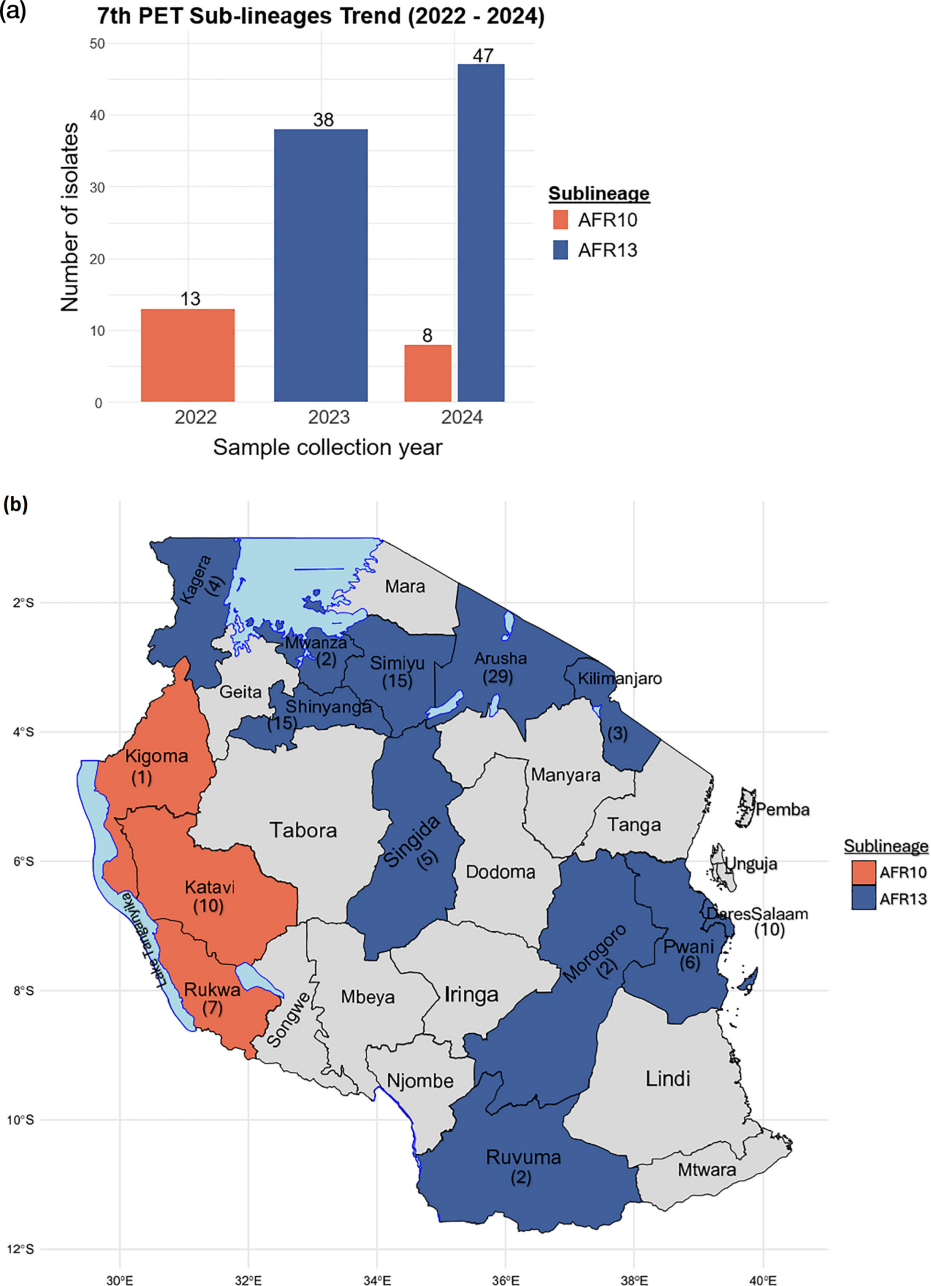
(**a**) A plot showing the trend of the *V. cholerae* lineages from 2022 to 2024. (**b**) A map of Tanzania with regional information of the circulating *V. cholerae*. Regions are coloured by the cholera sublineage lineages. T10 are found to be in regions around Lake Tanganyika. Grey-shaded regions indicate areas for which no *V. cholerae* isolates were available for sequencing in the NPHL biorepository during the study period.

Demographic characteristics of cases associated with sequenced isolates are summarized in Table 2 for the 119 samples. Sex information was available for 107 samples, of which 46 (38.7%) were from males and 61 (51.3%) from females; sex data were missing for 12 (10%) samples. Age stratification data was also captured and children aged 0–14 years accounted for 18 (15.2%) cases, young adults aged 15–34 years for 49 (41.2%), adults aged 35–54 years for 26 (21.8%), individuals aged 55–74 years for six (5%) and those aged ≥75 years for one case (0.8%). Age information was missing for 19 (16%) samples. Fig. 2a depicts a visual trend of sublineage frequency of the referred isolates for retesting and storage in NPHL across 2022–2024. There has been an overall increase in the number of AFR13 isolates over the study period compared to AFR10 isolates. Fig. 2b is a map of Tanzania highlighting the overall geographic coverage of the archived sequenced samples with *V. cholerae* with their sublineage status.

**Table 2.** Demographic and MLST characteristics of sequenced isolates (*N*=119). A summary of sublineage statistics includes only the samples with ST69 isolates (*N*=109)

Variable	Case (%)
***Sex***	
*Male*	*46* (*38.7*)
*Female*	*61* (*51.3*)
*Not indicated*	*12* (*10.0*)
***Age groups***	
*0–14*	*18* (*15.2*)
*15–34*	*49* (*41.2*)
*35–54*	*26* (*21.8*)
*55–74*	*6 (5.0*)
*75+*	*1* (*0.8*)
*Not indicated*	*19* (*16.0*)
***MLST***	
*ST69*	*109* (*91.6*)
*ST559*	*1* (*0.8*)
*ST947*	*1* (*0.8*)
*Unknown*	*8* (*6.8*)
**Isolate sublineages by year (*N*=109**)	
***2022***	
*AFR10*	*13*
*AFR13*	*0*
***2023***	
*AFR10*	*0*
*AFR13*	*38*
***2024***	
*AFR10*	*8*
*AFR13*	*47*
***Missing***	
*AFR10*	*1*
AFR13	2

### Cholera multi-locus sequence typing and transmission sublineages

MLST identified three sequence types among the 119 confirmed *V. cholerae* isolates. The vast majority (*n*=109, 91.6%) belonged to ST69, the predominant sequence type globally associated with the seventh El Tor pandemic lineage (7 PET). Single isolates of ST559 and ST947 (non-7PET) were also detected, while eight isolates could not be typed due to incomplete sequence coverage. Only ST69 isolates were used for downstream analyses, as ST69 represents the current pandemic strain of cholera.

Among the 109 ST69 isolates, transmission lineage assignment revealed that 22 (20.2%) belonged to the T10/AFR10 sublineage and 87 (79.8%) to the T13/AFR13 sublineage. Clear geographic stratification was observed: All AFR10 isolates were from regions around Lake Tanganyika (Katavi, Rukwa and Kigoma), while AFR13 isolates were distributed across other geographical zones including Northern (Arusha, Kilimanjaro), Eastern (Dar es Salaam, Pwani, Morogoro), Central (Dodoma, Singida), southern zone (Ruvuma) and Lake zones (Mwanza, Kagera, Shinyanga, Simiyu, Kigoma) (see Fig. 2b).

### Genotypic and phenotypic antimicrobial resistance profiles

**Fig. 3.**
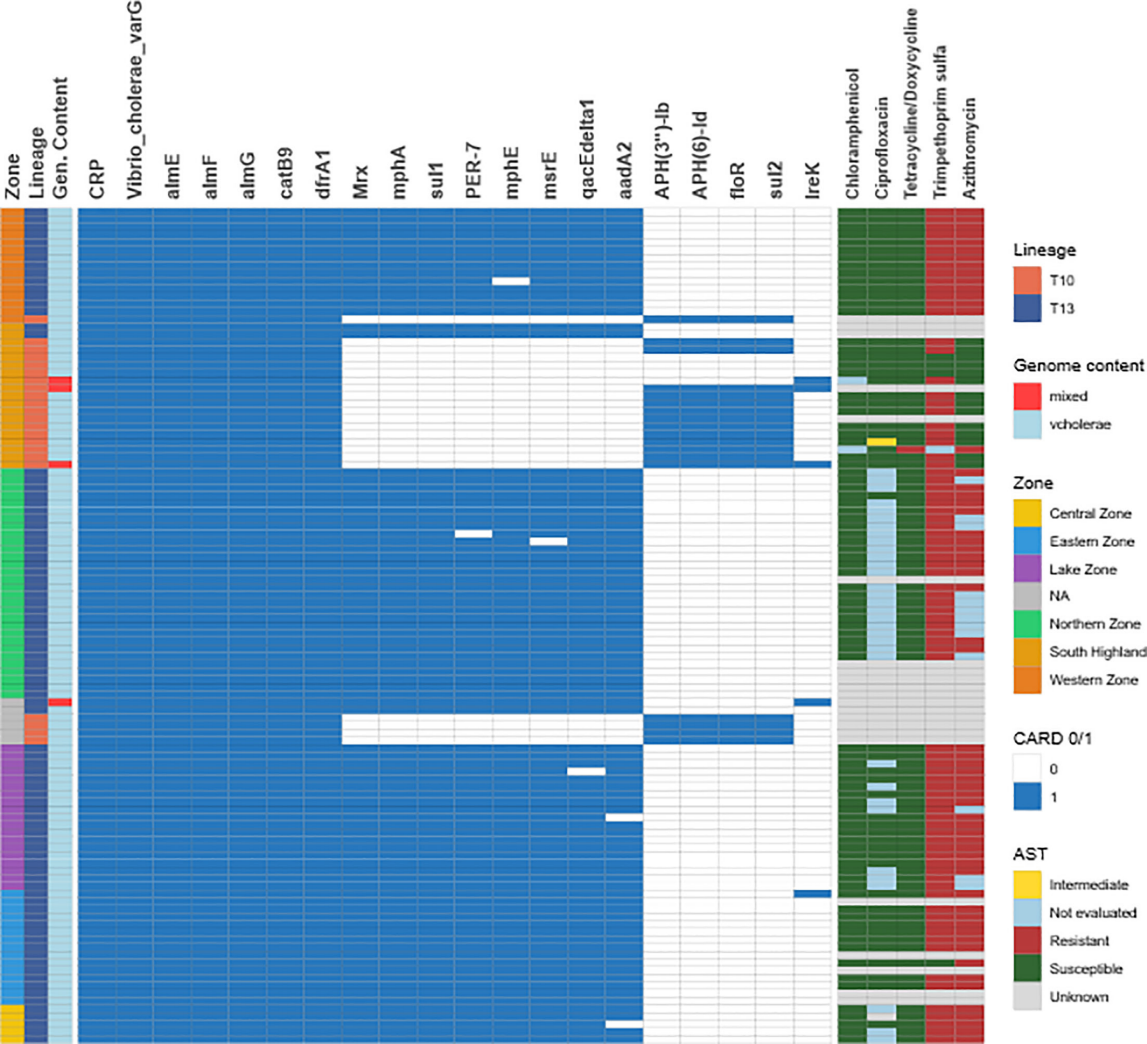
Distribution of AMR genes by lineage, phenotypic resistance and zone. The right panel shows the phenotypic resistance confirmed through antimicrobial susceptibility test (AST) results conducted at NPHL for each isolate.

All 109 ST69 isolates carried AMR-associated genes, with individual genomes containing between 7 and 22 detected resistant determinants (Fig. 3). The most common resistance-associated genes included *catB9* (chloramphenicol resistance), *sul1* in AFR13 and *sul2* in AFR10 isolates (sulfonamide resistance), *dfrA1* (trimethoprim resistance) and *varG* (streptomycin resistance). The *mph(A)* and *mph(E)* (linked to macrolide resistance), and *qacEdelta* genes (resistance to quaternary ammonium compounds), were specifically observed predominantly in AFR13 isolates while none of the genes were detected in the AFR10 sequences. Extended-spectrum β-lactamase (ESBL) gene *blaPER-7* was identified in nearly all AFR13 isolates except one, based on CARD and ResFinder annotations. Tetracycline resistance genes (*tetA, tetB, tetC*) were not detected in any isolate.

Phenotypic antimicrobial susceptibility testing (AST) showed that most isolates were susceptible to chloramphenicol, ciprofloxacin and tetracycline/doxycycline. Resistance to trimethoprim-sulfamethoxazole (SXT) was observed in the majority of both AFR10 and AFR13 isolates tested. All AFR13 isolates were resistant to azithromycin, whereas most AFR10 isolates remained susceptible. Isolates resistant to SXT carried sul1/sul2 and dfrA1 genes. AFR13 isolates were phenotypically resistant to azithromycin and harboured the macrolide resistance-associated genes mph(A) and mph(E), whereas AFR10 isolates were largely susceptible to azithromycin and lacked both genes. No tetracycline resistance genes (tetA, tetB or tetC) were detected in any isolate.

### Virulence gene profiles and plasmid markers

**Fig. 4.**
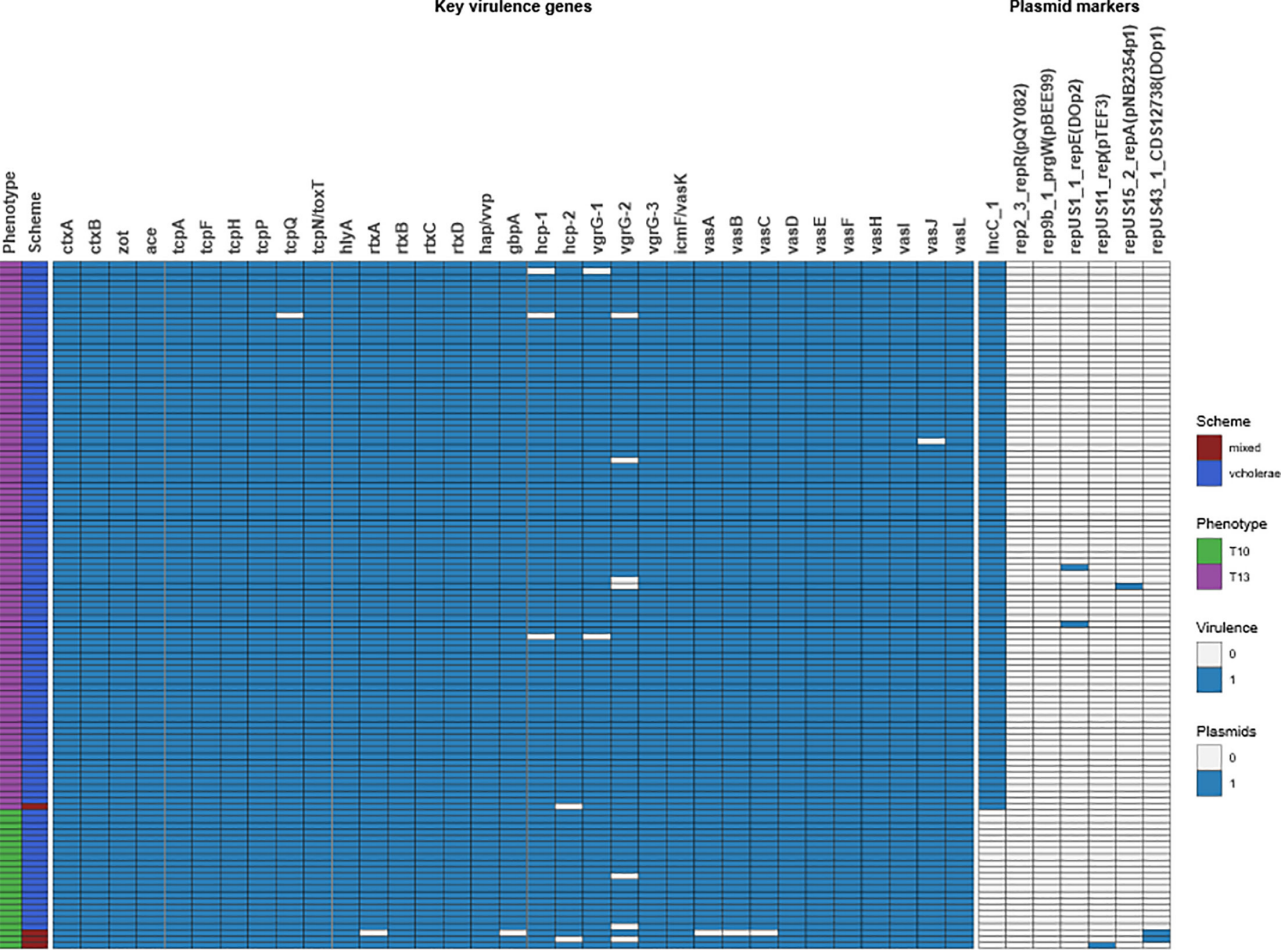
Heatmap summarizing the key virulence genes and plasmid markers found in the ST69 *V. cholerae* isolates.

All ST69 isolates carried the core virulence genes essential for cholera pathogenesis, including cholera toxin genes (*ctxA, ctxB*), toxin-coregulated pilus gene (*tcpA*) and genes encoding accessory virulence factors (*zot, ace, hlyA, rtxA*) (Fig. 4). Cholera toxin subunit B allele distribution showed clear sublineage association as AFR13 isolates carried *ctxB7* (characteristic of the third pandemic wave with major outbreaks that occurred in Yemen and Haiti [37, 38]), while AFR10 isolates predominantly harboured *ctxB1* (early third wave marker). The isolates carried the hallmark components of Vibrio Pathogenicity Island 1 (*VPI1*) which include the complete tcp operon (*tcpA-tcpT*) and the *acfA-acfD* colonization factors detected through the VFDB. The isolates also possessed VPI2, as demonstrated by the presence of *nanH*, a key marker of the sialic acid utilization cluster that is a defining feature of this ∼57 kb genomic island [39].

The presence of the core virulence genes, such as the *ctxA, ctxB* and components of the *VPI1* and *VPI*2 in our study, supports that the isolates uniformly harbour the canonical pathogenicity islands and toxigenic phage elements associated with epidemic *V. cholerae* and therefore possess the full complement of genetic determinants required for colonization and cholera toxin-mediated disease. *VPI1* is known to encode the toxin coregulated pilus (*TCP*), the essential intestinal colonization determinant and receptor for *CTXΦ* infection in epidemic *V. cholerae* [40].

### Plasmid reconstruction results

**Fig. 5.**
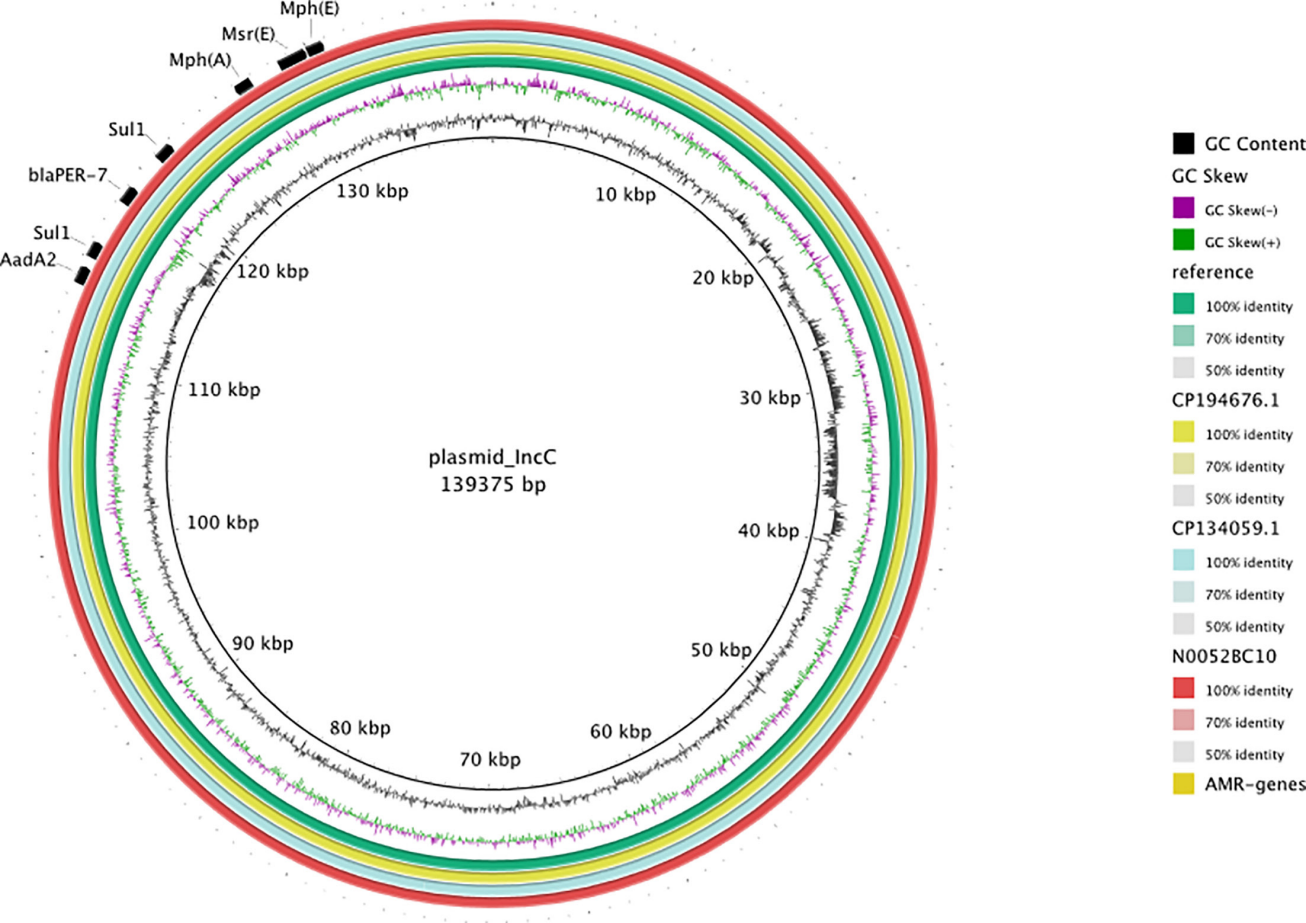
A plasmid reconstructed from sample N0052BC10.

All sequenced AFR13 isolates carried an *IncC* plasmid (N0052BC10) of 139 kb, which contained *sul1, blaPER-7, aadA2, mph(A)* (-ve strand), *msr(E)* and *mph(E)* resistance genes similar to pVCMLK181 constructed by Imoli *et al*. from the 2022 Kenyan outbreak [41] (Fig. 5). This N0052BC10 plasmid has displayed 100% nucleotide sequence identity to CP194676.1 and CP134059.1, which were previously identified in highly drug-resistant AFR13 strain outbreaks.

### Phylogenetic analysis

**Fig. 6.**
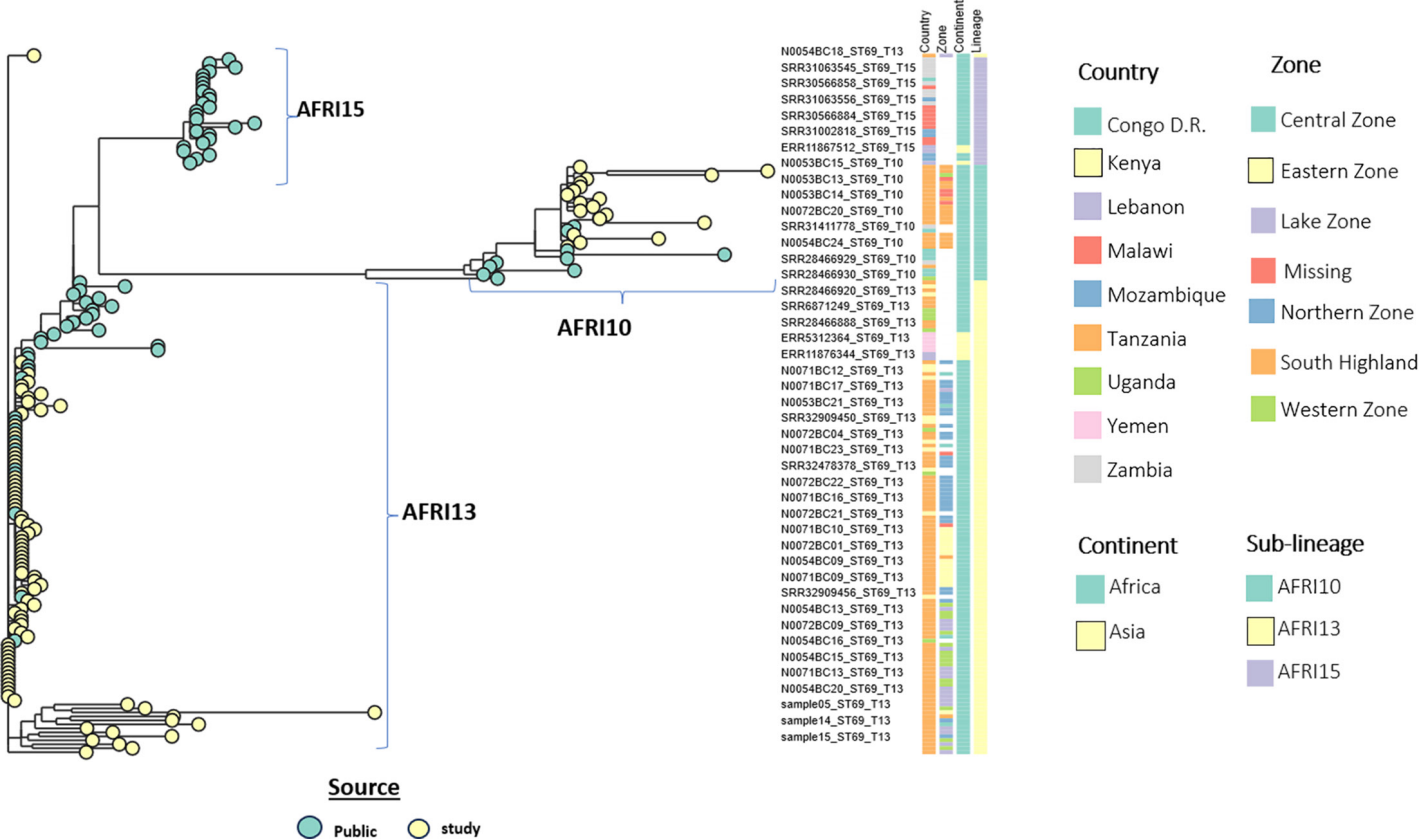
Phylogenetic tree showing Tanzania sequences from this study (yellow tips) alongside background public sequences from other countries (blue tips) and previously sequenced Tanzanian isolates. All Tanzanian sequences were under the AFR10 and AFR13 clades.

Maximum-likelihood phylogenetic analysis based on core genome SNP positions revealed two distinct clades corresponding to the AFR10 and AFR13 sublineages. The phylogenetic tree showed clustering by geographic regions and sublineages, with two major clades separating Tanzania AFR10 and AFR13 isolates as outlined in Fig. 6.

Within the AFR10 clade, isolates from Katavi and Rukwa formed small slightly independent clusters, suggesting local transmission in the respective regions. The AFR13 clade was more diverse, with isolates from multiple regions showing genetic relatedness. Isolates from Arusha, Dar es Salaam, Shinyanga and Simiyu formed several subclusters differing by 10–25 SNPs.

**Fig. 7.**
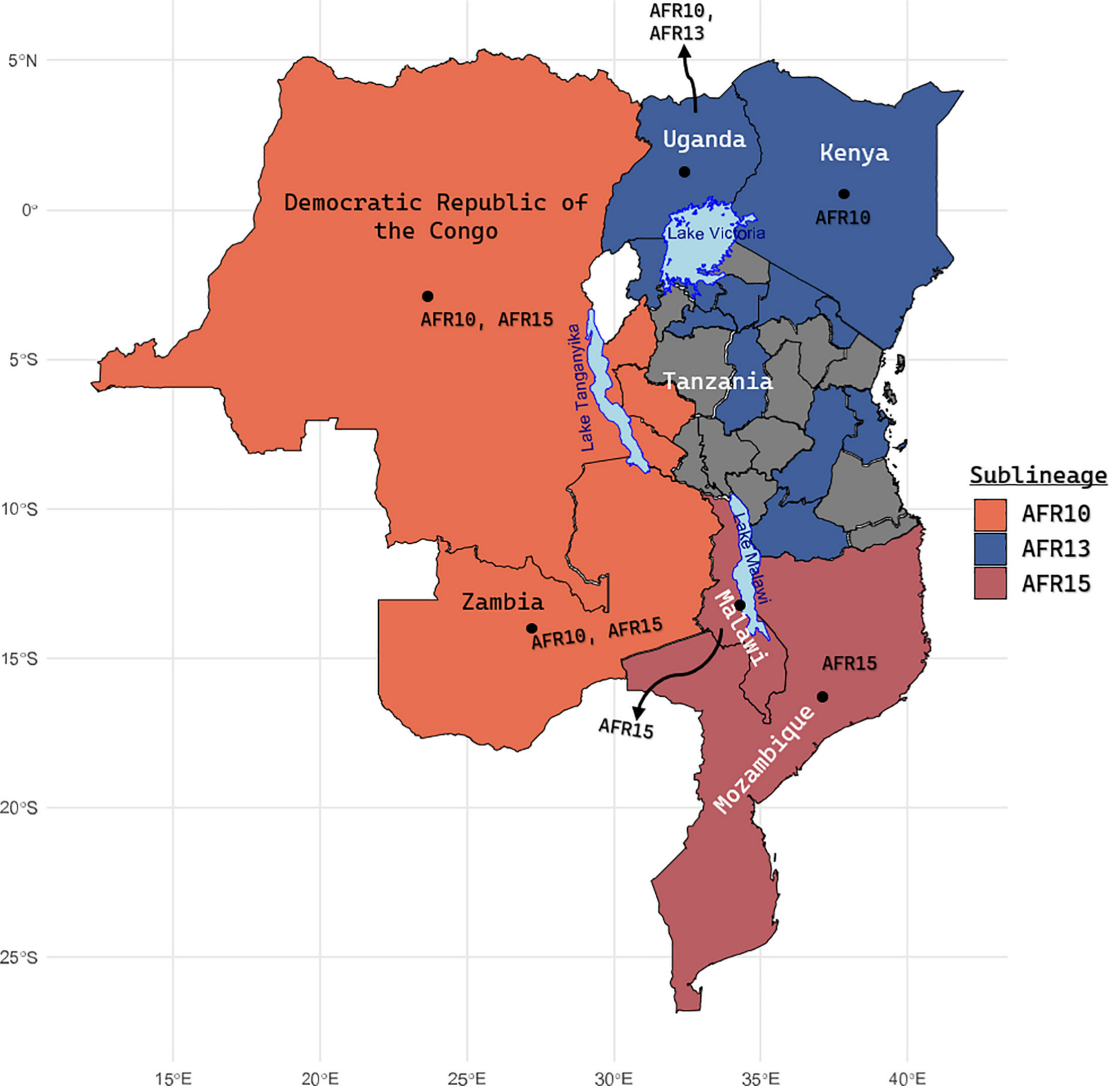
A map showing Tanzania alongside other neighbouring countries. Countries are coloured with sublineages shared with Tanzania isolates. Some countries have more than one sublineage as highlighted below the country name. Uganda sequences had both AFR10 and AFR13 as Tanzania.

**Fig. 8.**
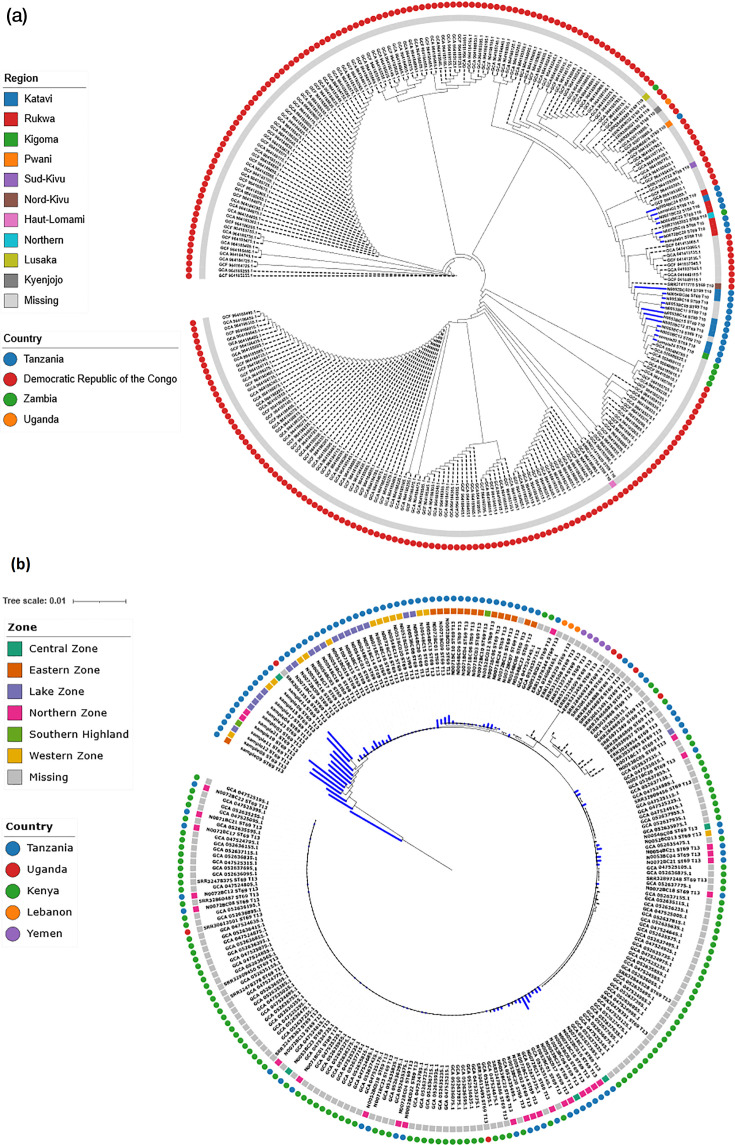
Sublineage-specific phylogenetic comparison. Branches with solid blue lines represent our study samples, whereas dashed black lines represent publicly available sequences. Panel (**a**) shows the AFR10 phylogenetic tree and panel (**b**) shows the AFR13 phylogenetic tree, each compared with publicly available sequences from neighbouring countries.

To better understand a snapshot of the sublineages comparison and distribution of the downloaded genomes in relation to our samples, we prepared a map (Fig. 7) with all the countries surrounding Tanzania. A summary of the identified sublineages is labelled below each country’s name, and neighbouring countries are coloured according to the similar sublineages they share with Tanzania. Further sublineage-specific phylogenetic comparisons with the neighbouring countries revealed that AFR10 isolates are related to isolates from countries bordering Lake Tanganyika. These isolates clustered with isolates from DR Congo, Zambia and Uganda, and interestingly, all isolates from Rukwa clustered closer with isolates from the northern region of Zambia, which borders Tanzania across Lake Tanganyika (Fig. 8a). Isolates from Katavi and Kigoma also clustered around Zambia and DR Congo. On the other hand, phylogenetic comparison of AFR13 revealed relatedness with isolates from Kenya, Uganda and Yemen to isolates from different zones in Tanzania (Fig. 8B).

## Discussion

This study presents a comprehensive genomic analysis of *V. cholerae* isolates collected during the cholera outbreaks in Tanzania between 2022 and 2024. The geographic distribution of sequenced isolates in our study largely corresponds to regions that have historically reported cholera cases or outbreaks in the country. However, the absence of sequence data in the other regions does not indicate the absence of cases in these areas. It reflects the retrospective nature of the isolate archive, including variation in outbreak occurrence, specimen referral, successful culture, shipment to NPHL, isolate storage conditions and viability at the time of retrieval. Despite these, the available genomic data provide valuable insights into the spatial distribution and circulation of cholera sublineages and can be used to enhance understanding of the geographic epidemiology of cholera in Tanzania.

With this data, we demonstrate that the *V. cholerae* isolates harboured the virulence markers responsible for causing outbreaks [37–40]. The outbreak-associated isolates were dominated by two 7PET sublineages, AFR10 and AFR13, both belonging to MLST ST69, with clear geographic and temporal structuring, although a small number of additional sequence types (ST559, ST947 and unknown ST) were also detected. Although AFR15 has been reported in neighbouring countries including Zambia, Mozambique and Malawi [42, 43], it was not detected among the isolates analysed in this study. This observation is consistent with findings reported by Kachwamba *et al*. who found no evidence of related strains with AFR15 isolates from Mozambique [44]. Nevertheless, the presence of AFR15 in neighbouring countries highlights the importance of continued regional genomic surveillance to detect potential future introductions.

From our results, we observed a spatial restriction of AFR10 in regions around Lake Tanganyika basin which follows a similar pattern reported since 1998 by Hounmanou *et al*. [45]. This distribution suggests long-term local persistence within a stable ecological niche, potentially supported by environmental reservoirs. Our findings are consistent with previous genomic studies demonstrating endemic circulation and microevolution of *V. cholerae* O1 in the Lake Tanganyika region over multiple decades [6, 15]. Furthermore, the close genetic relatedness between Tanzanian AFR10 isolates and strains reported from neighbouring countries is consistent with regional circulation within the Lake Tanganyika basin, although the available metadata are insufficient to infer direct person-to-person cross-border transmission [16]. This observation is further supported by our phylogenetic analysis, in which isolates from the Tanzanian regions of Rukwa, Kigoma and Katavi clustered with isolates from the DR Congo and the northern region of Zambia, which borders Tanzania through Rukwa. Together, these findings are compatible with the persistence of AFR10 within the Lake Tanganyika ecosystem. However, the available epidemiological and geographic metadata are insufficient to distinguish between transmission driven by a shared environmental reservoir and direct cross-border human transmission.

In contrast, AFR13 displayed a broader geographic distribution among the sequenced isolates and became increasingly dominant from 2023 to 2024 referred isolates during the study period. This pattern is consistent with earlier genomic evidence showing that AFR13 emerged after 2014 and progressively displaced earlier lineages in Tanzania [6]. Unlike AFR10, which remained largely restricted to the Lake Tanganyika basin, AFR13's wide distribution is likely due to regional interaction activities and environmental adaptability rather than specific hotspot regions. The genomic features, such as more resistance markers and the presence of a plasmid, in AFR13 in this study likely indicate a greater chance for rapid transmission compared to AFR10. The detection of AFR13 also in Kenya and Uganda [16, 41, 46] further indicates successful regional circulation of this sublineage, warranting further investigations on its transmission dynamics.

Despite the widespread distribution of AFR13 observed in subsequent years, no AFR13 isolate was identified among the sequenced samples in 2022. The reasons for this observation remain unclear and should be interpreted with caution given the limited number of available isolates in that period. Possible explanations include limited availability of viable isolates in 2022, stochastic sampling of outbreak-associated isolates, changes in health-seeking, surveillance and specimen referral patterns and enhanced hygiene practices such as handwashing during the period following the COVID-19 pandemic [47–49], which may have influenced cholera transmission dynamics and case detection. However, these explanations remain hypotheses because the available data do not allow formal evaluation.

Notably, AFR13 isolates increased in geographic distribution after 2022, suggesting progressive expansion across Tanzania. The widespread circulation of AFR13 across diverse ecological settings may indicate enhanced persistence and transmission under varying environmental conditions, although further studies are needed to confirm this hypothesis. Consistent with previous reports in other countries [50, 51], cholera outbreaks have increasingly occurred during dry seasons in Tanzania, contrasting with the traditional association with rainy periods. This pattern may reflect a combination of bacterial, environmental and socioeconomic factors, including drought-induced reliance on potentially contaminated water sources such as wells, dams and surface water bodies. Sublineage-specific differences were also observed in this study. AFR13 isolates more frequently carried *qacEdelta,* which has previously been reported to be associated with resistance to multiple antibiotics and antiseptics [52], and macrolide resistance genes [(*mph(A), mph(E)*], whereas AFR10 isolates had none. AMR patterns observed in our isolates are consistent with previous reports of 10 kb deletions in SXT elements characteristic of AFR13 strains in Tanzania [6]. The observed accumulation of AMR genes among AFR13 isolates which have been linked to macrolide resistance [*mph(A), mph(E)*] and disinfectant resistance (*qacEdelta*), further suggests continued evolutionary adaptation under antimicrobial and control pressures. Similar evolutionary trajectories have been observed in large epidemic settings, where extensive antimicrobial use facilitated the emergence and spread of multidrug-resistant clones [37]. In contrast, the absence of tetracycline resistance genes and susceptibility to the antibiotic across all isolates likely supports the continued effectiveness of doxycycline-based treatment regimens which is consistent with clinical guidance and recent regional observations [11, 53]. Moreover, it is important to note that, despite the presence of *catB* gene which is usually labelled as associated with chloramphenicol resistance, all isolates were phenotypically sensitive when tested to the respective antibiotic in this study. This finding is consistent with a study by Weill *et al.* [12], which found it to be a silent chloramphenicol acetyl transferase gene (reported in the article supplementary text) and hence cannot be considered responsible for chloramphenicol resistance when identified by whole-genome sequences of *V. cholerae* El Tor.

Overall, our findings are consistent with broader regional genomic evidence indicating that cholera transmission in East and Central Africa is shaped by both long-standing endemic reservoirs such as around Lake Tanganyika basin [45] and episodic regional dissemination of highly transmissible lineages. Previous studies have demonstrated strong genetic similarity between clinical and environmental isolates from lake ecosystems, supporting a role for aquatic environments in long-term persistence and seasonal amplification [6]. Comparable dynamics have been documented in Asia, where plankton-associated ecosystems sustain cholera transmission over time [54]. The persistence of AFR10 around Lake Tanganyika corresponds with the patterns reported elsewhere in the African Great Lakes region, while the expansion of AFR13 across multiple countries reflects a continent-wide trend of lineage replacement following repeated introductions of fitter seventh-pandemic variants [6, 12, 13, 45]. Evidence from multi-country genomic analyses further indicates that such lineages can persist across national boundaries over extended periods, facilitated by population mobility and shared ecological corridors [46].

Generally, under public health perspectives, adaptability of *V. cholerae* to more stable, highly transmissible and resistant sublineages is of concern. Recent large-scale outbreaks, including the catastrophic epidemic in Malawi driven by an imported ST69 clone (AFR15), illustrate the public health consequences of regional connectivity [43] when surveillance is not properly coordinated. The detection of disinfectant resistance genes in our isolates further raises concerns for infection prevention and control, particularly in cholera treatment centres, as similar genetic profiles have been linked to reduced biocide susceptibility in major outbreak settings [37].

Several limitations should be considered when interpreting these findings. First, isolates were obtained through outbreak response and referral to the NPHL biorepository rather than through systematic population-based sampling. The dataset therefore overrepresents cases and regions where isolates were collected, successfully cultured, transported, stored and recovered, and it cannot be used to estimate regional prevalence or incidence of *V. cholerae.* Second, the retrospective use of archived isolates limited the availability and completeness of epidemiological metadata, including detailed exposure histories, travel information and clinical outcomes, thereby restricting fine-scale reconstruction of transmission chains. Third, although AST results were available for key antibiotics, disinfectant efficacy testing was not performed; therefore, detection of *qacEdelta* should be interpreted as a genomic signal requiring phenotypic follow-up rather than proof of reduced disinfectant susceptibility. Fourth, sequencing was performed using Oxford Nanopore technology, which is well suited for rapid outbreak genomics but has a higher per-base error profile than short-read sequencing. We therefore relied on genome-quality thresholds and lineage-level analyses to reduce the effect of platform-associated errors. Finally, some genomes could not be fully characterized because of variable sequence quality, although this did not affect the primary lineage-level conclusions.

This study fills a critical gap in cholera surveillance in Tanzania by providing the first nationwide genomic assessment of circulating *V. cholerae* lineages since 2017, during a period of renewed cholera resurgence in Africa. Through integrating genomic, geographic and resistance data, it offers actionable evidence to inform treatment guidelines, outbreak preparedness and geographically targeted interventions. The findings support continued use of doxycycline while highlighting emerging risks associated with macrolide and disinfectant resistance. More broadly, this work underscores the necessity of sustained national and cross-border genomic surveillance, integrated with environmental monitoring, as a cornerstone of cholera control and elimination efforts in Tanzania and the wider East and Central African region.

## Conclusion

Whole-genome sequencing of *V. cholerae* isolates from cholera outbreaks in Tanzania between 2022 and 2024 identified two 7PET transmission lineages, T10/AFR10 and T13/AFR13, predominantly belonging to ST69, with distinct geographic distributions. AFR10 was restricted to regions bordering Lake Tanganyika, whereas AFR13 was widely distributed across multiple zones and dominated in later outbreak years. Phylogenetics analysis reveals several neighbouring countries having AFR15, highlighting the importance of continued regional surveillance to detect and prevent potential future introductions of a third sublineage into Tanzania. All MLST ST69 isolates carried core virulence genes, and AFR13 harboured multiple AMR determinants. Macrolide and ESBL resistance genes, as well as the *qacEdelta* gene, were predominantly detected in AFR13 isolates. The *qacEdelta* gene has been reported by other studies to be associated with reduced susceptibility to multiple antibiotics and antiseptics; however, phenotypic testing was not investigated in our study. In our study isolates, tetracycline resistance genes were absent, and phenotypic susceptibility to tetracycline/doxycycline was maintained across tested isolates, thus supporting current treatment guidelines; however, resistance signals on other antibiotics warrant continued monitoring. Enhanced genomic surveillance capacity, integration of environmental surveillance and strengthened regional collaboration will be essential in advancing towards cholera elimination goals in Tanzania and the broader East African region.

## Supplementary material

10.1099/mgen.0.001813 Supplementary Material 1.

10.1099/mgen.0.001813 Supplementary Material 2.

10.1099/mgen.0.001813 Supplementary Material 3.

## Abbreviations

AMR: antimicrobial resistance
AST: antimicrobial susceptibility test
BA: blood agar
CARD: Comprehensive Antibiotic Resistance Database
ESBL: Extended-spectrum β-lactamase
MLST: multi-locus sequence typing
NPHL: National Public Health Laboratory
7PET: seventh pandemic El Tor
QC: quality check
SNPs: Single-nucleotide polymorphisms
SXT: sulfamethoxazole
TCBS: thiosulfate-citrate-bile salts-sucrose
TCP: toxin coregulated pilus
VFDB: Virulence Factor Database
VPI: Vibrio Pathogenicity Island
