# Supplementary material for: Genomic epidemiology reveals geographically structured co-circulation of AFR10 and AFR13 Vibrio cholerae lineages driving cholera outbreaks in Tanzania (2022–2024)
Source: Microb Genom. 2026 Aug 18;12(8):001813. doi: 10.1099/mgen.0.001813 (PMC13485274; doi:10.1099/mgen.0.001813)
Supplement: Supplementary file 1 [file mgen-12-01813-s001.pdf]

```
##########################################
#Assembly with Bactopia pipeline. This performs major pre-processing steps
including QC check and assembly.

# Prepare sample sheet from FASTQ files
bactopia-prepare \
   --path "$fastq_dir"" \
   --fastq-ext .fastq.gz \
   --species Vibrio_cholerae \
   --genome-size 4800000 \
   > "$output_dir/samples.txt"

# Run Bactopia on all assemblies
if [-s "$output_dir/samples.txt" ]; then
   bactopia \
      --samples "$output_dir/samples.txt" \
      --species Vibrio_cholerae \
      --cpus $CPUS \
      --outdir "$output_dir/bactopia_runs" \
else
   echo "No FASTQ samples detected in $fastq_dir"
fi


##########################################
#Multi-Locus Sequence Type (MLST) identification

for file in ${INPUT_DIR}/*.fasta
do
   # Get base filename and extension
   basename=$(basename "$file" .fasta)

   echo "Running MLST for $basename..."

   # Run mlst
   result=$(mlst "$file")

   # Extract ST (second column)
   ST=$(echo "$result" I awk '{print $2"_ST"$3}')

   # If ST is not found, skip
   if [[ -z "$ST" ]]; then
      echo "No ST found for $basename, skipping..."
      continue
   fi

   newname="${basename}_ST${ST}.fasta"

   echo "Renaming to $newname"
   cp "$file" "${INPUT_DIR}/${newname}"
```

done

##
#Sublineage identification using USHER.
#We developed the Patho2Clade tool for a rapid offline sub-lineage identification. See the GitHub repository link in the methods section for a detailed workflow.

```
usher -i "$TREE" \
        -v "$OUTDIR/snps.vcf" \
        -o "$OUTDIR/placement.pb"
```

##
#AMR, Virulence genes & Plasmid detection (default parameters used)

```
# List of databases
databases=("card" "resfinder" "ncbi”, “vfdb”, “plasmidfinder”)

# Looping through ST69.fasta genomes
for genome in "$input_dir"/*ST69.fasta; do
   base=$(basename "$genome" .fasta)

   for db in "${databases[@]}"; do
      echo "Running Abricate on $genome with $db..."
      abricate --db "$db" "$genome" > "$output_dir/${base}_${db}.tab"
   done
done
```

##
#Phylogenetic analysis.

```
# Run Prokka for annotation.
# Reference annotation file: reference_gb="references/ncbi_dataset/data/
GCF_000006745.1/genomic.gbff"

      prokka \
        --kingdom Bacteria \
        --genus Vibrio \
        --species cholerae \
        --prefix "$short_prefix" \
        --locustag "$short_prefix" \
        --outdir "$output_dir/$sample" \
        --cpus $THREADS \
        --compliant \
        --force \
        --proteins "$reference_gb" \
        "$tmp_fasta" \
```

```
        > "$output_dir/${sample}_prokka.log" 2>&1

#Core alignment with panaroo

panaroo \
    -i "${gff_files[@]}" \
    -o "$panaroo_out" \
    -t "$THREADS" \
    -a core \
    --clean-mode strict

#Gubbins: recombination analysis

run_gubbins.py \
  --prefix "gubbins_run" \
  --threads $THREADS \
  "$core_alignment"

#SNPs Extraction with SNP-sites

snp-sites -c "$filtered_alignment" > "$analysis_out/core_snps.fasta"
snp-sites -o "$analysis_out/core_snps.vcf" "$filtered_alignment"

#maximum likelihood tree with IQ-TREE
iqtree2  -s "$analysis_out/core_snps.fasta" -m MFP -nt AUTO -bb 1000 -pre
"$analysis_out/iqtree"
```
